# Investigating the Emulsifying Mechanism of Stereoisomeric Sugar Fatty Acyl Molecular Gelators

**DOI:** 10.1021/acs.langmuir.3c03274

**Published:** 2024-06-27

**Authors:** Sai Sateesh Sagiri, Malick Samateh, George John

**Affiliations:** † Department of Chemistry and Biochemistry, the City College of New York, 160 Convent Avenue, New York, New York 10031, United States; ‡ Doctoral Program in Chemistry, the City University of New York, Graduate Center, New York, New York 10016, United States

## Abstract

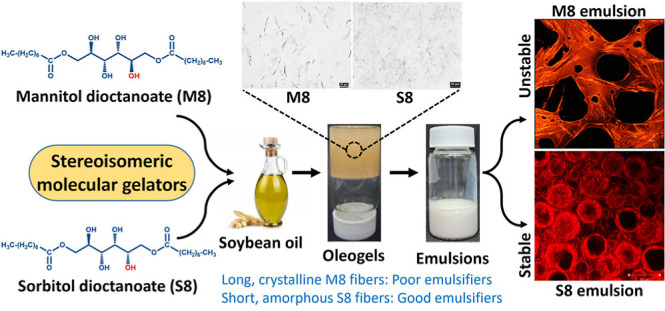

The emulsifying mechanism of supramolecular stereoisomeric sugar fatty acyl molecular gelators was evaluated. In-house-synthesized mannitol dioctanoate (M8) and sorbitol dioctanoate (S8) were tested. The stereoisomeric difference between the sugar groups significantly affected the gelation and emulsifying properties of the gelators. M8 and S8 formed oleogels at 2 and 3.5% (w/v) and emulsified water up to 30 and 60% (v/v), respectively. Microscopy showed that the gelator fibers are at the W/O interfaces, demonstrating a solid particle or network mode of stabilization. The long fibers of M8 were unable to completely encompass the water droplets, resulting in poor emulsification. Small, hair-like fibers of S8 showed better emulsification. When sunflower wax (SFW, 1% w/v) was added as a coemulsifier, synergetic action between the wax and S8 improved the stability of emulsions. Such synergy was not seen between SFW and M8, henceforth emulsion stability was not improved. This study proved that a subtle stereoisomeric difference at the molecular level can greatly alter the supramolecular and emulsifying properties of sugar-fatty acyl compounds.

## Introduction

In the dominion of emulsion science, the quest for effective emulsifiers that combine stability, biocompatibility, and sustainability is always challenging. Sugar-fatty acyl derivatives are one such class of emulsifiers designed as an alternative to conventional fat and amino acid-derived emulsifiers. A variety of sugar-fatty acyl derivatives, such as alkyl polyglycosides, sorbitan esters, and sucrose esters, are developed as nonionic emulsifiers.^1−3^ Although several sugar-fatty acyl derivatives exist, their mode of emulsifying mechanism is nearly the same. In general, the sugar derivative is solubilized in either water or oil, and then the immiscible liquids are emulsified by forming micelles at the oil–water interface.^3,4^ However, to the best of our knowledge, solid particle or interfacial network-based emulsification from sugar-fatty acyl derivatives has never been seen. In fact, natural emulsifiers such as hydrocolloids,^5^ phospholipids,^6^ waxes,^7^ and proteins^6^ stabilize the emulsions by adsorbing at the interfacial juncture in the form of crystals, fibers, and solid particles.

**Figure 1:**
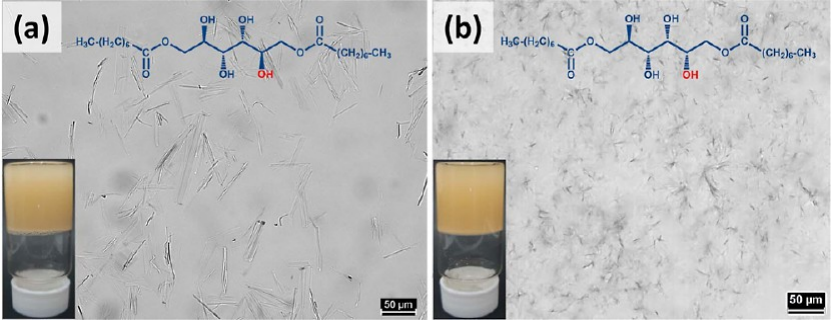
Microstructure of (a) M8 and (b) S8 oleogels.

Inspired by natural emulsifiers, our research group has synthesized sugar-fatty acyl derivatives using biocompatible raw materials. Our lab has pioneered the development of a facile, lipase-mediated esterification method to synthesize sugar-fatty acyl derivatives.^8−10^ The synthesized sugar-fatty acyl derivatives had shown supramolecular properties by self-assembly in organic solvents and vegetable oil. This kind of supramolecular nature facilitated the use of sugar-fatty acyl derivatives as molecular gelators while forming oleogels. In our previous studies, we have shown their use as fat replacers^10^ and wax-free lip balms.^11^ In all the cases, sugar-fatty acyl derivatives were used as oil structuring agents, and their emulsifying ability was never explored. In this study, stereoisomeric sugar alcohol derivatives, namely, mannitol dioctanoate (M8) and sorbitol dioctanoate (S8), were used for this purpose. The molecular structures of S8 and M8 are similar except for the stereoisomeric difference between precursor sorbitol and mannitol moieties (Figure 1). This difference had shown a significant impact on the physical, thermal, and mechanical properties of their resulting oleogels.^12^ The effect of such a stereoisomeric molecular difference on the emulsifying properties of supramolecular compounds is intriguing. Henceforth, a detailed investigation was conducted in this study to evaluate the emulsifying nature of the molecular gelators. Previously, oleogelators such as fatty acids,^13^ fatty alcohols,^14,15^ monoglycerides,^16^ waxes,^17,18^ gums,^6^ shellac resin,^19^ and biopolymeric derivatives^20^ were used to prepare emulsions. To the best of our knowledge, this is the first of its kind to report the emulsifying nature of stereoisomeric molecular gelators.

The main objective of this study is to identify the emulsifying mechanism of molecular gelators (S8 and M8) and to demonstrate their emulsifying efficiency. For this, emulsions were prepared using a simple three-component (water, vegetable oil, and molecular gelator) system without any surfactants or emulsifiers. A detailed microscopy involving brightfield and fluorescent microscopic studies was conducted to prove the emulsification mechanism. To study the effect of a stabilizer on the emulsification behavior of molecular gelators, we used sunflower wax (SFW).

## Materials and Methods

### Materials

Analytical grade (purity >98%) sorbitol, mannitol, and vinyl octanoate were purchased from TCI America (Portland, OR). SFW was a kind gift from Cargill USA. Novozym 435 (lipase from *Candida antarctica*) was provided by Novozymes North America (Franklinton, NC). Hexanes and acetone were purchased from Fisher Scientific (Suwannee, GA). Soybean oil was purchased from a local supermarket. Double-distilled water was used whenever it was necessary.

#### Preparation of Molecular Oleogels

Accurately weighed molecular gelators were added to soybean oil in glass vials, which were then heated above their melting point (M8: 126 °C and S8: 78 °C) under continuous agitation.^12^ After the complete dissolution of the gelators, the vials were allowed to incubate and cool to room temperature. The formation of oleogels was checked by the vial inversion method.

#### Preparation of Emulsions

Molecular oleogel-based emulsions were prepared using 0.5 to 5% (w/v) gelators in soybean oil with varying water volumes from 10% (v/v) to 65% (v/v). This was done by following two approaches, namely pre- and postcrystallization methods. Precrystallized emulsions were prepared by adding water (dropwise) to the solidified or precrystallized oleogel. For the preparation of postcrystallized emulsions, molten oleogels were emulsified by the dropwise addition of water. Both kinds of emulsions were prepared using 0.5 to 5% (w/v) gelators in soybean oil with varying water volumes from 10% (v/v) to 65% (v/v).

In both regimes, emulsification was carried out by homogenizing the mixture at 8000 rpm for 15 min using UltraTurrax T20 (IKA–Werke GmbH & Co. KG, Germany). While preparing wax-containing emulsions, the same procedure was followed except that 1% (w/v) SFW was added to the gelator-oil mixture prior to heating.

#### Microscopy

The microstructures of oleogels, emulsions, and emulgels (emulsion gels) were deciphered by viewing them under a bright field or fluorescent microscope, Leica DM 2000 LED (Leica Microsystems, Germany). In the fluorescent microscopy, water-soluble dyes, green-color-yielding fluorescein, and red-color-yielding rhodamine B, were used. Molecular gelators were dyed by adding pure gelators to water containing rhodamine B, followed by agitation at room temperature overnight at 200 rpm using an orbital shaker incubator. The gelators were filtered by using Whatman filter paper and then dried in a hot air oven at 37 °C for 24 h.

The particle size distribution (PSD) of emulsions was analyzed by using ImageJ software. To quantify the distribution, ∼500 droplets were measured from the micrographs of each emulsion. The size of droplets (volume–surface mean diameter, *D*_3,2_) and their polydispersity (span value) were determined as follows 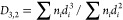
where *n*_*i*_ is the number of droplets having *d*_*i*_ diameter, and
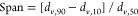
where *d*_v,90_, *d*_v,10_, and *d*_v,50_ are the corresponding diameters of particles at 90, 10, and 50% cumulative volume, respectively.

## Results and Discussion

### Preparation of Oleogels

In house-synthesized sugar-fatty acyl derivatives (as described in our previous study)^21^ were used to decipher their gelation and emulsification efficiencies. The gelation ability of M8 and S8 was confirmed by inverting the vials after 24 h of their dissolution in soybean oil (shown as insets in Figure 1). The stereoisomeric difference between the sugar groups of gelators has significantly affected their gelation efficiency. The minimum gelation concentrations (MGC) of M8 and S8 were found to be 2% (w/w) and 3.5% (w/w), respectively, whereby the lower MGC value for M8 indicates its higher gelation efficiency. The self-assembled fibrillar networks (SAFiNs) of the two gelators were quite distinct, where M8 formed long, needle-like fibers, and S8 formed small, hair-like fibers (Figure 1). In addition to the fiber size and shape, their orientation was also found to be different. While M8 fibers are solitary in nature, S8 fibers are aggregated in clusters (Figure 1). Yang et al. showed that 12-hydroxy stearic acid derivatives with different alkyl chain lengths can form a variety of SAFiNs with variable length and diameter.^22^ In the current study, stereoisomeric discrepancies between the gelators resulted in distinct self-assembly patterns, which in turn affected the gelation efficiency.

### Preparation of Emulsions

#### Effect of the Water Addition Mode

Emulsions were prepared by the following two approaches. In the first, water was added dropwise to the 5% (w/v) M8 oleogel, and in the other, oleogel was added to water. In both cases, the added water concentration was maintained at 50% (v/v) relative to that of the bulk phase. In either case, water droplets were dispersed in the oleogel continuous phase, evident from the fluorescent microscopy (Figure S1). However, the mode of water addition affected the droplet size. The presence of smaller and relatively uniform droplets was seen when water was added to the oleogels. Henceforth, the dropwise addition of water to the oleogel was followed for the rest of the study.

#### Gelator Precrystallization vs Postcrystallization

Emulsification behavior of oleogelators was further assessed by adding water (50% v/v) to either structured oleogels (precrystallized gelators before water addition) or molten gelator–oil mixture (postcrystallization of gelators after water addition). The microarchitecture of both emulsions looks alike, wherein the gelator fibers are dispersed throughout the continuous phase but more at the oil–water interfaces (indicated by arrows in Figure S2a). No significant difference in size or shape of the gelator fibers was seen in both regimes. However, a size difference in the dispersion was noticed between M8 and S8 emulsions, where larger droplets were formed in the former compared to the latter (Figure S2). Though the efficiency is different, gelators followed solid network stabilization in both regimes, where SAFiNs are aggregated at the oil–water interface. Previously, the influence of pre- and postcrystallization procedures was studied by preparing W/O emulsion gels using medium- and long-chain diacyl glycerols (DAGs).^23^ The postcrystallization procedure resulted in smaller fat crystals of DAGs, which eventually yielded more stable emulsions. On the contrary, M8 and S8 have formed stable emulsions in both procedures. This can be attributed to the difference in the crystallinity of gelators. DAGs formed fat crystals; on the other hand, the sugar-fatty acyl derivatives formed less crystalline fibrous networks, which are not susceptible to the applied shear during emulsification. The poor crystallinity of S8 and M8 SAFiNs was confirmed in our previous study.^21^

Since no significant difference was observed, the postcrystallization regime was followed in further experiments. The rationale is to allow the interactions between water and gelators to occur at the molecular level prior to their crystallization. This kind of interaction could enhance the surface activity of molecular gelators on water droplets. Since this study does not involve incorporating any additional surfactants, enhancement of the surface activity of molecular gelators in their molten state would improve the emulsification.

#### Effect of Gelator Concentration

**Figure 2:**
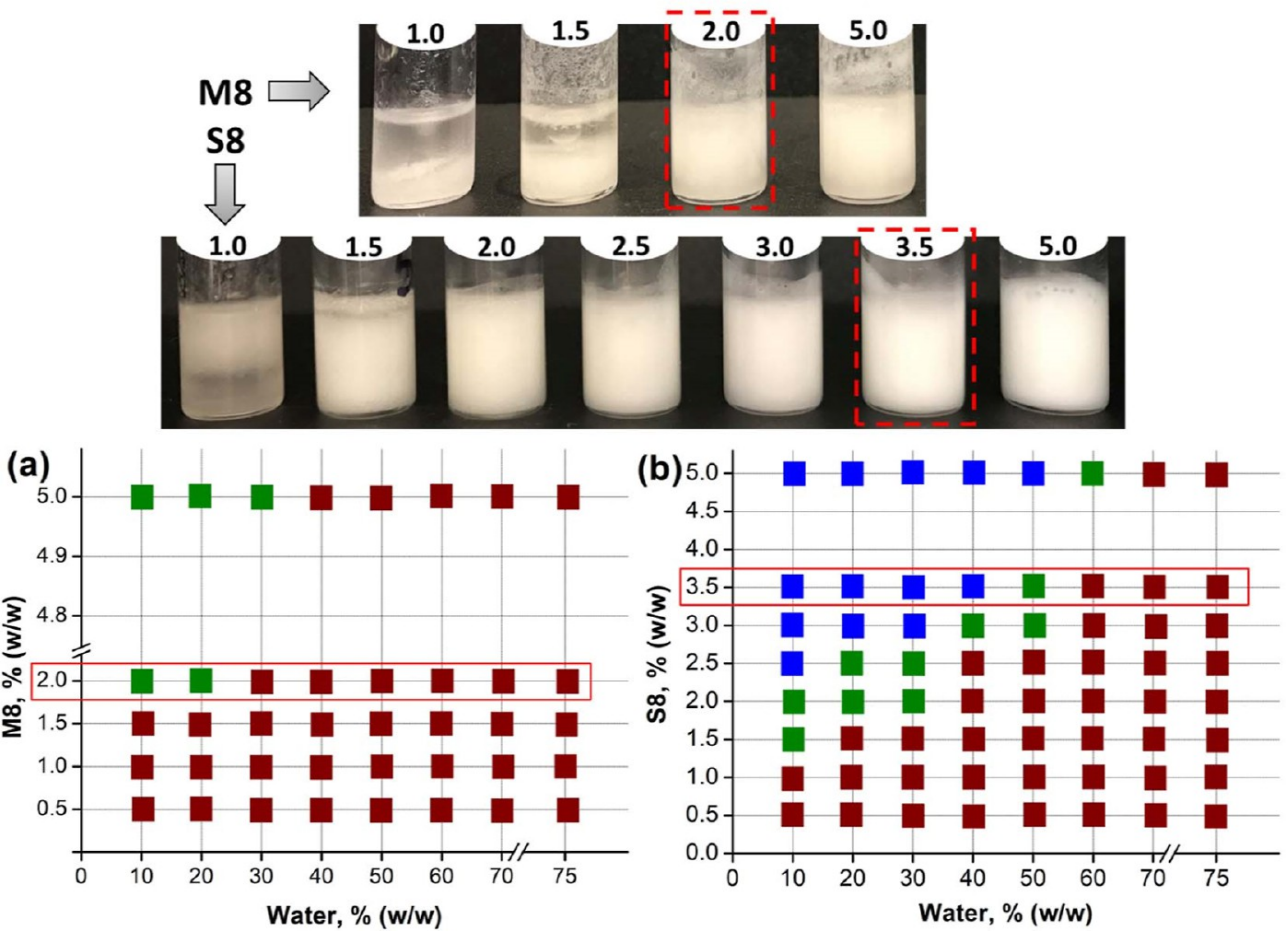
Graphical representation of the changes in gelator and water concentration in (a) M8 and (b) S8 emulsions. Vials at the top of the figure are representative emulsions with varied gelator concentrations and a constant water concentration, 50% (w/v). In the graph, brown boxes: unstable emulsions; green boxes: stable emulsions up to one week; blue boxes: stable emulsions for more than four weeks.

**Figure 3:**
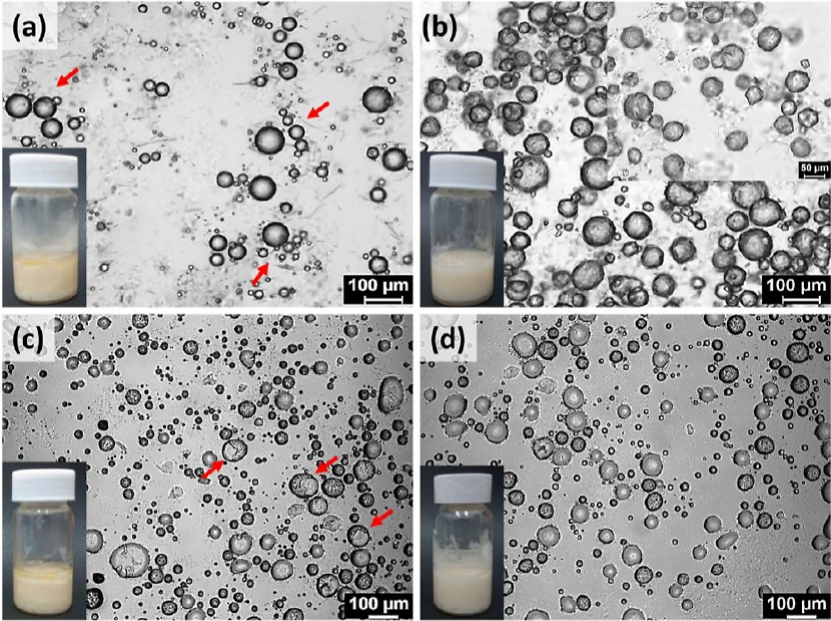
Microscopic images of M8 (top, a,b) and S8 (bottom, c,d) gelators, each showing unstable (left) and stable (right) emulsions.

The concentration of both gelators varied from 0.5 to 5% (w/v), and water was maintained at 50% (v/v) of the oil. Prior to this, the water volume was varied from 10 to 75% (v/v) at a particular gelator concentration. The stability of the obtained emulsions is graphically presented in Figure 2a,b. Based on this and to understand the effect of gelator concentration, a constant water volume was maintained at 50% (v/v). Immediate phase separation was observed at lower gelator concentrations, but emulsion formation started happening near MGC (highlighted with red tracing or boundaries around the vials and boxes in Figure 2). M8 did not form any emulsions until MGC was reached, but S8 formed emulsions even below its MGC. At MGC, M8 emulsions phase separated within a day, and S8 emulsions remained stable for 2–3 days. To understand the phenomenon, microscopy was performed on both unstable and stable emulsions. M8 unstable emulsions showed sparsely dispersed SAFiNs and droplet aggregation (indicated by arrows in Figure 3a). On the contrary, stable M8 emulsions showed closely packed water droplets with interfacial fibers (highlighted region of Figure 3b). In the case of S8, some of the droplets were not entirely covered by gelator fibers in unstable emulsions (indicated by arrows in Figure 3c), but such partial covering was not seen in stable emulsions (Figure 3d). Lack of sufficient interfacial networks was responsible for the instability in both emulsions.

Based on the emulsified water volume, M8 emulsions were labeled as M8-10, M8-20, and M8-30, and S8-10 to S8-60 for S8 emulsions. The gelator concentration was maintained at 5% (w/v) for the labeled M8 and S8 samples. The composition of the emulsions is given in Table S1. M8 formed emulsions up to a 30% dispersion volume (Figure 2a), whereas S8 formed stable emulsions up to 60% (Figure 2b). The presence of water hindered the extension of M8 fibrous networks and led to liquid emulsions (Figure S3). On the contrary, S8 formed semisolid emulsion gels (emulgels), which are stable for at least three months, except for S8-60 (Figure S4). This can be due to the retention of S8 fibrous networks at the interfaces and in the continuous oleogel phase of the emulsions.

#### Effect of SFW

SFW (melting point of SFW: 75–78 °C)^24^ and gelators were heated together prior to the emulsification. The rationale for choosing wax as the coemulsifier is due to its similar emulsifying mechanism as that of gelators. Wax stabilizes emulsions by forming interfacial crystals and by increasing the viscosity of the oil.^25,26^ Based on this fact, we predicted that wax would assist the gelators during emulsification.

##### M8 Emulsions

The addition of SFW enhanced the water emulsification to 40% (w/w) (Figure S3), without significantly prolonging the stability of the emulsions. This indicates that SFW had little effect on strengthening the M8 interfacial fibrous networks. In general, waxes possess weak surface activity on water droplets but contribute to the stability of emulsions by increasing the oil viscosity. Use of surface-active compounds such as glycerol monooleate and lecithin would have improved the emulsification.^27,28^ Since the objective of this study was to establish the emulsifying nature of molecular gelators, surface-active emulsifiers were not used. Moreover, wax concentration was also restricted to 1% (w/v) so as to limit its role as coemulsifier and not supersede the gelator performance.

##### S8 Emulsions

The addition of SFW did not enhance the emulsification of water beyond 65% (v/v) (Figure S4) but prolonged its stability. For e.g., S8W-60 and S8W-65 stability was extended for another week. This indicates that SFW is in synchrony with the S8 fibrous network during emulsification.

### Microscopic Analysis

#### M8 Emulsions

**Figure 4:**
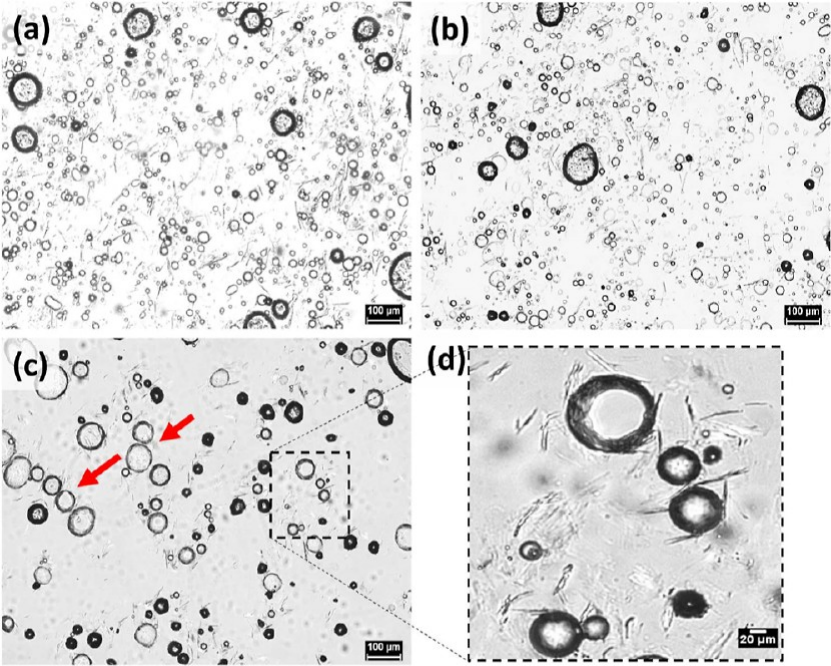
BFM images of (a) M8-10, (b) M8-20, and (c) M8-30 and (d) magnified portion of M8-30.

Polydisperse droplets and randomly distributed fibers were seen in the M8 emulsions (Figure 4). The long fibers of M8 aggregated around the water droplets but were not enclosed entirely (Figure 4d). This kind of poor surface activity exposed water to oil, leading to the formation of flocs (shown with arrows in Figure 4c). The inefficiency of M8 fibers as emulsifiers can be attributed to their length and needle-like nature. However, this kind of fiber provides better gelation (evident from MGC), higher crystallinity, and thermal stability to oleogels.^12,29^ This implies that M8 is an excellent oleogelator but a poor emulsifier. Lu et al. also showed the poor emulsification of long fibers.^30^ The addition of SFW also did not alter the microstructure, as polydispersity and long fibers were seen in M8W emulsions too (Figure S5). Though SFW crystals were not distinctive (due to their low concentration (1% w/v)), their effect was discernible from the delayed phase separation.

#### S8 Emulsions

**Figure 5:**
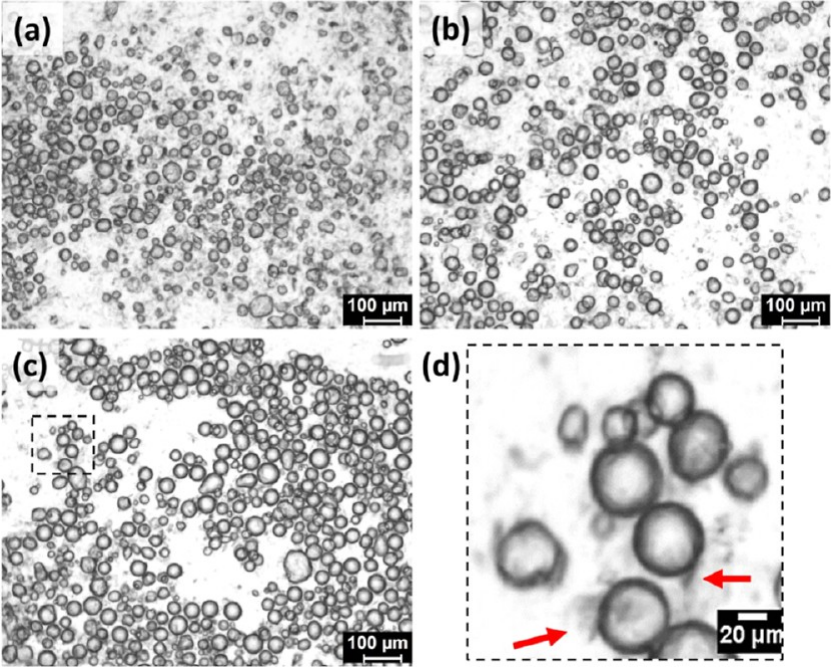
BFM images of (a) S8-30, (b) S8-40, (c) S8-50, and (d) magnified portion of S8-50.

Relatively uniform and spherical water droplets were seen in the S8 emulsions (Figure 5). Unlike M8, S8 fibers were not distinct. The dark patches around the droplets correspond to aggregated S8 fibers (indicated by the arrows in Figure 5d). This kind of dense orientation facilitated the formation of stable emulsions, including the concentrated (S8-50) and highly concentrated emulsions (S8-60). A subtle stereoisomeric change resulted in a radical change in the emulsifying efficiency of gelators. The improved emulsification can be ascribed to the lower crystallinity of S8 fibers. X-ray diffraction studies in our previous work determined that hair-like fibers of S8 are amorphous and needle-like fibers of M8 are crystalline in nature.^21^ As amorphousness facilitates more degrees of freedom, S8 fibers are oriented effectively to form a dense network at the interfaces. The interfacial steric stabilization was further improved upon the addition of SFW as highly concentrated emulsions (S8W-65) were formed, along with the enhanced stability of S8W emulsions. This kind of steric stabilization was also shown by lecithin-ceramide gelator crystals in oleogel-based surfactant-free emulsions.^31^ Being surface-active, ceramides (waxy lipids) formed interfacial crystals while stabilizing the oleogel-in-water emulsions. Similarly, the fatty acyl component of gelator fibers and SFW facilitated the interfacial stability of oleogel-in-water emulsions. S8 or S8W emulsions showed less polydispersity, but a significant increase in size was noticed when dispersion volume was higher (Figure S7).

#### Size Distribution Analysis

PSD in emulsions was expressed as Gaussian plots and cumulative functions (cumulative particle diameter distribution, CPDD). For this, volume–surface mean droplet diameter (*D*_3,2_) was calculated.

#### M8 Emulsions

**Figure 6:**
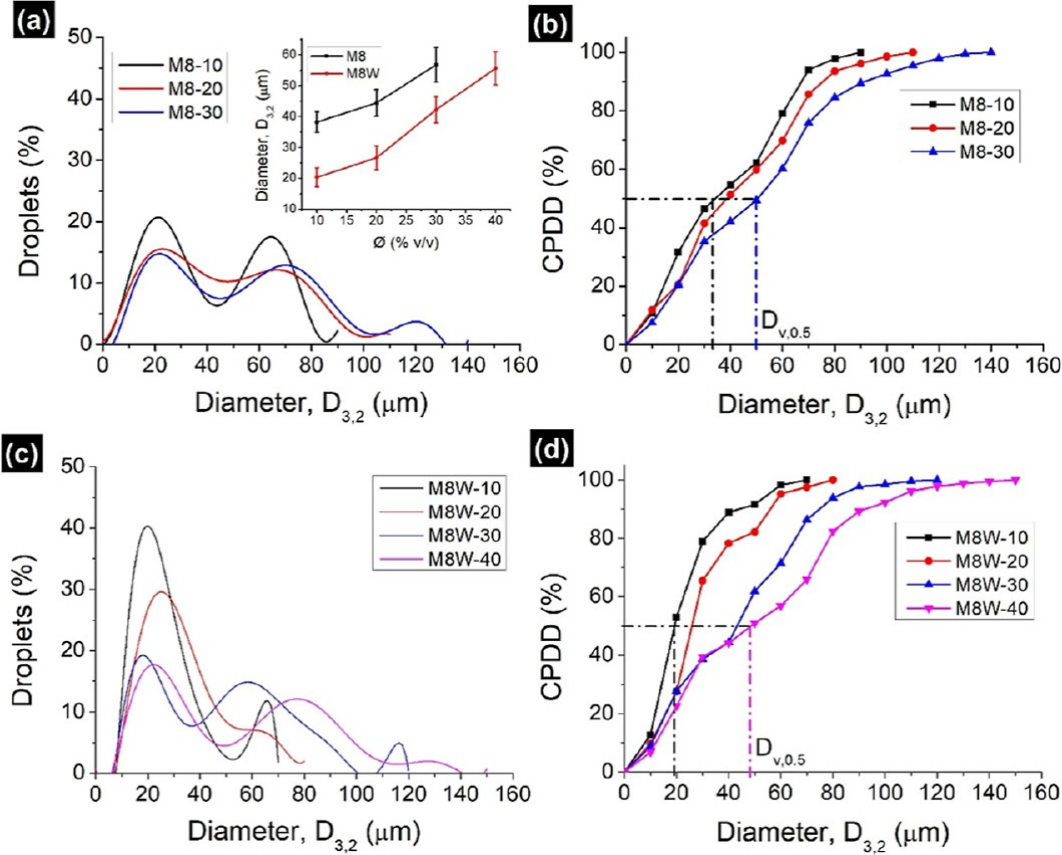
Size distribution analysis of (a,b) M8 and (c,d) M8W emulsions.

Gaussian plots of M8 and M8W emulsions showed a bimodal and trimodal distribution of droplets at 10–40, 50–80, and 120–130 μm (Figure 6a,c). Right-shift of curves with the increase in Ø corresponds to the increase in droplet diameter (inset in Figure 6a). A sudden increase in *D*_3,2_ was noticed in both M8 and M8W emulsions when Ø was raised from 20 to 30% (v/v). This implies the poor emulsifying nature of M8 at 30% (v/v) water. The effect of wax also seems negligible, as it did not improve the emulsification. Median droplet diameter (*D*_v,0.5_)—the particle size of 50% of the total droplet population—was measured from CPDD graphs. Both *D*_3,2_ and *D*_v,0.5_ followed the same pattern, with a sudden increase in diameter after 20% (v/v) of dispersion (Figure 6b,d and Table S2). This result was also supported by another parameter, the “span” value, an indicator of polydispersity. Based on span values (Table S2), all the emulsions are polydisperse, but they suddenly increased after 20% (v/v) of the dispersion. All PSD parameters (*D*_3,2_, *D*_v,0.5_, and span) point out the poor emulsifying nature of M8 fibers and the insignificant role played by the SFW in emulsions.

#### S8 Emulsions

**Figure 7:**
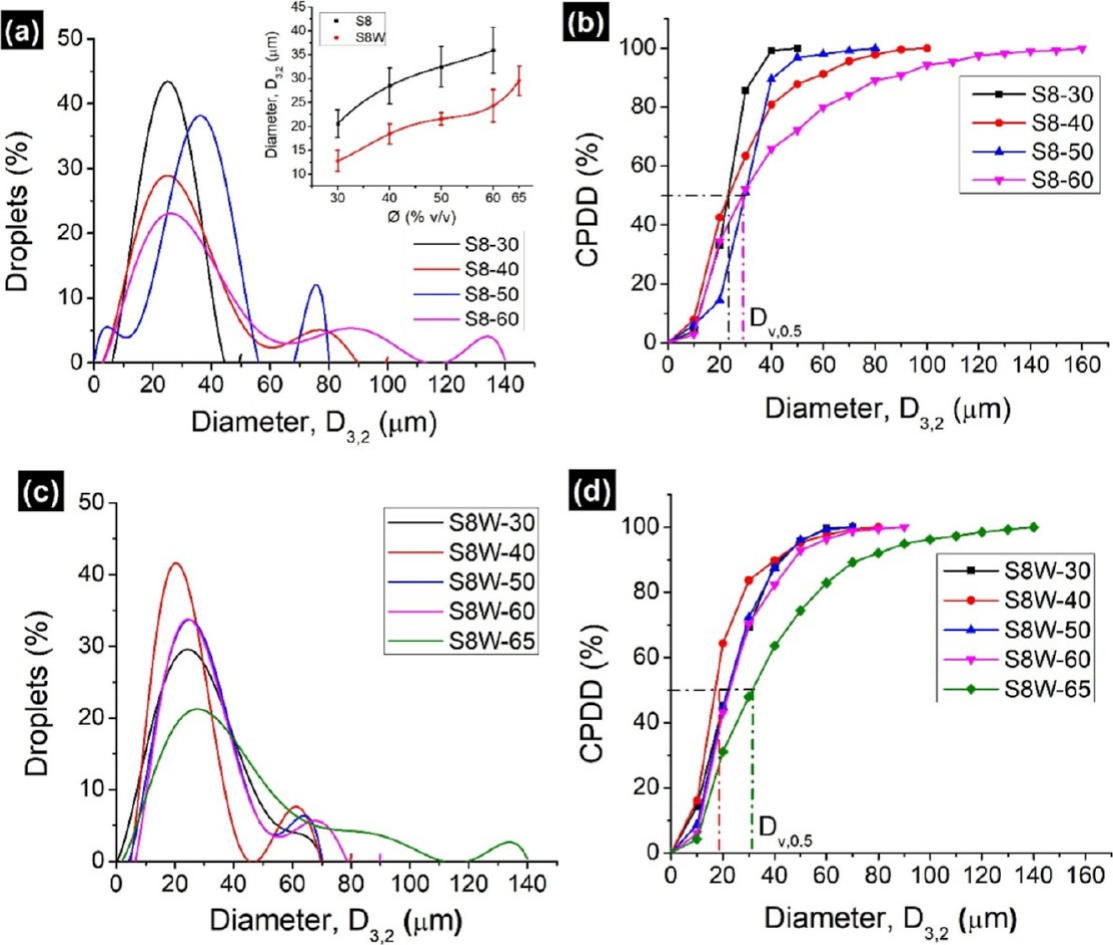
Size distribution analysis of (a,b) S8 and (c,d) S8W emulgels.

Compared to M8 Gaussian plots, a narrow size distribution was seen in both S8 and S8W emulsions, with the majority of droplets in the range of 10–40 μm (Figure 7a,c). Increase in Ø created bimodal (S8-40 and S8-50) trimodal (S8-60) and multimodal (S8W-65) distribution. A linear increase in diameter with Ø was seen in all emulsions except S8W-65, where an exponential increase occurred (inset in Figure 7a). Similarly, the *D*_v,0.5_ values of all S8 and S8W emulsions are close to each other, except S8W-65 (Figure 7b,d and Table S2). Besides the increased size, concentrated emulsions (S8-60, S8W-60, S8W-65) showed high polydispersity (evident from span values, Table S2) and destabilized after two weeks. The remaining S8 and S8W emulsions were highly stable. Among all, S8-50 and S8W-50 were chosen as the model emulgels for time-dependent analysis. They were periodically tested via microscopy and found to be stable for three months.

Microscopic and PSD data showed that the droplet size increased during storage. Signs of aggregation and flocs were seen on the 60th and 90th days, respectively (Figure S9). The majority of droplets are in the range of 10–50 μm, but their percentage decreased significantly (Figure S10a,c). The increase in size and polydispersity were evident from the *D*_v,0.5_ and Span values (Figure S10b,d and Table S2). The right-shift of CPDD curves was prominent in S8-50 but not in S8W-50 indicating that droplet coalescence was hindered in S8W-50. This can be due to the synergetic action between S8 fibers and SFW crystals and the increased viscosity of the oleogel continuous phase (evident from the rheology studies). Although there is some coalescence, S8-50 and S8W-50 remained stable after three months of storage at room temperature. Being stable for three months is remarkable for surfactant-free oleogel-based emulsions. Recently, lecithin/stearate and lecithin/sorbitan tristearate-based oleogels formed stable emulsions without any stabilizers.^32^ Similarly, lecithin/ceramide oleogel-based emulsions showed no signs of destabilization up to two weeks after preparation.^31^ In another study, a high percentage of monoglycerides (10–35% w/w) were able to stabilize oleogel-based nanoemulsions for 10 months.^33^ In all these studies, a surface-active compound (e.g., lecithin) was used to perform as an emulsifier and to assist in oil structuring. However, in this study, no such compound was used, and the surface/emulsifying activity of novice molecular gelators (M8 and S8) was explored. The obtained results suggest that the emulsifying nature of the studied molecular gelators is on par with that of standard emulsifiers.

### Emulsification Mechanism

#### Wettability Test

Since this study was designed to understand the emulsification efficiency of molecular gelators, their amphiphilic nature was expected to facilitate interfacial activity. The phase behavior of molecular gelators was understood by adding them to water and incubating them for 1 h at room temperature. While M8 remained on top of the water, S8 was seen as suspension. To enhance the gelator–water interaction, the samples were vortexed (at 3000 rpm) for 30 s. No significant change in the dispersion behavior of gelators was seen (indicated by the arrows in Figure S11). This simple test confirms that M8 is hydrophobic by nature. Although S8 and M8 possess the same HLB (hydrophilic lipophilic balance) value according to the Griffin scale, their discrete phase behavior can only be attributed to the stereoisomerism of sugars considering the structural similarity of the appended fatty acid chains. A subtle difference in the orientation of a single hydroxyl group affected both gelator–gelator and gelator–water interactions and resulted in SAFiNs with diverse morphology and solubility, respectively. This is evident from the molecular dynamics’ simulation studies.^34,35^ The simulation studies showed that the structural configurations of mannitol and sorbitol are influenced by the presence of water. Despite structural similarities and negative hydration behaviors, their specific interactions with water are quite different. In water, mannitol adopts a planar zigzag configuration and sorbitol adopts a bent-chain configuration.^34^ Due to the adopted configuration, sorbitol produces a larger disruption of the water surface and leads to its higher solubility than mannitol at room temperature.^36^

#### Effect of Postcrystallization

As mentioned before, the postcrystallization regime was followed to enhance the gelator–water interactions at the molecular level and to facilitate the adsorption of gelators at the water–oil interphase. Another significance of this regime is that the addition of water to the molten gelator-oil mixture facilitates in situ quench-cooling of gelator fibers.^37^ Quench-cooling crystallization helps in the formation of smaller fibers, which are better emulsifiers than large fibers.^37^ However, the crystallization behavior of S8 and M8 was not changed here, as their size (Figures 4 and 5) remained the same as that in oleogels (Figure 1). Because of the prevailing hydrophobicity and manifestation of long fibers, M8 failed to encompass and stabilize the dispersed water effectively. In contrast, small S8 fibers showed superior emulsification. Similar results were also seen in emulsions stabilized with monoacyl glycerols and microcrystalline fat particles.^38,39^

#### Effect of Shear

Though quench-cooling did not affect the size of fibers, it affected their growth as networks. When oleogels were subjected to emulsification, a lack of fiber networks became apparent in M8 emulsions (Figure 4) but less conspicuous in S8 emulgels (Figure 5). The addition of water and rotational shear hindered the growth of the fibers into networks. To further understand this, oleogels were prepared under shear. In the absence of water, the molten gelator–oil mixture was sheared under the same conditions (8000 rpm for 15 min) as those of emulsification. Unlike conventional oleogels, the gels underwent shear flow when inverted (Figure S12). Though the physical state was changed, the fibers under shear were similar to those of typical S8 and M8 oleogels (Figure S12). This suggests that although the gelators were crystallized into fibers under the shear, further extension into fibrous networks was hindered. Lack of gelator networks affected the stability of the emulsions, especially M8 emulsions.

#### Effect of Wax

**Figure 8:**
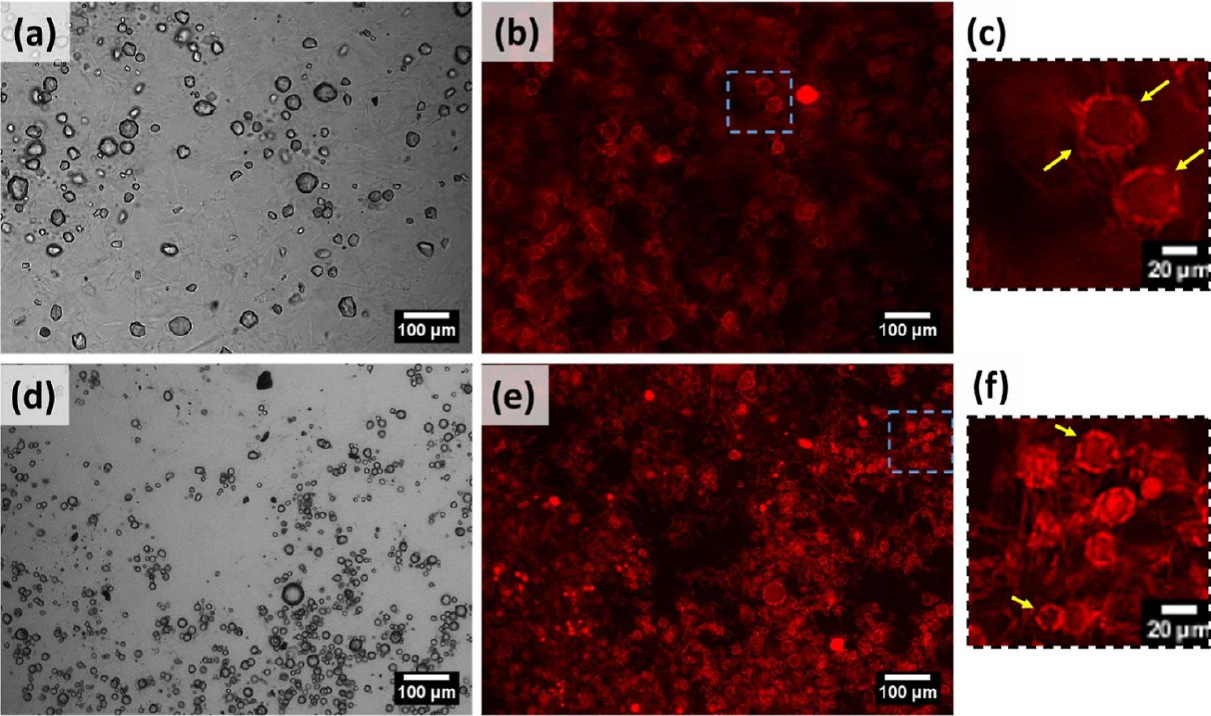
BFM and FM images of (a–c) M8-30 and (d–f) M8W-30.

**Figure 9:**
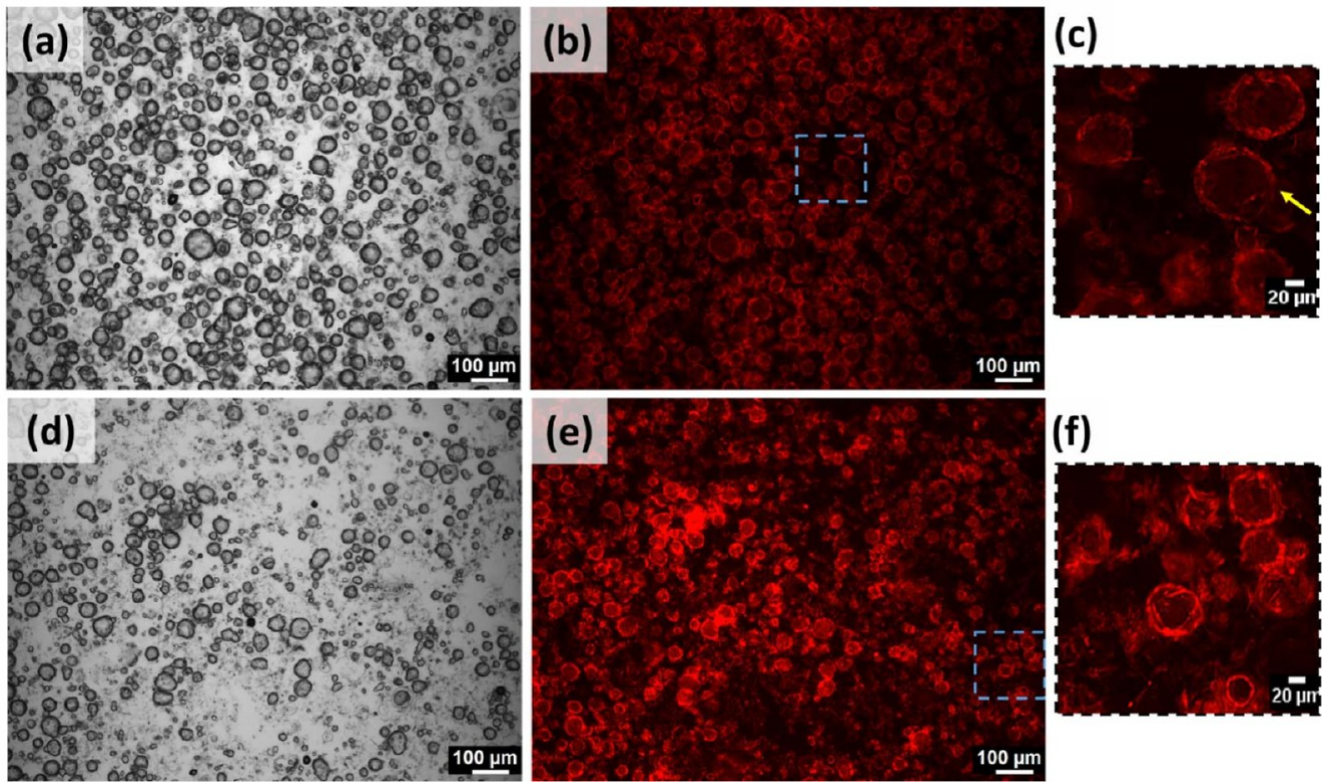
BFM and FM images of (a–c) S8-50 and (d–f) S8W-50.

The rationale to choose SFW as a coemulsifier is because of its melting point, which matches S8 (78 °C). For better comparison, SFW was added to M8 too; moreover, it is very difficult to add a natural wax that matches the melting point of M8 (120 °C). It was hypothesized that since water offers a nucleation surface for wax crystallization, water droplets could be stabilized by the interfacial wax particles along with the gelator fibers. The synergetic effect of the wax crystals/surfactant^40,41^ and solid particles/surfactant^42^ is not uncommon in stabilizing complex emulsions. Because of melting point similarities, the cocrystallization of S8 and SFW created a cooperative synergetic effect and enhanced the emulgels stabilization. However, a similar synergetic effect is not possible in M8 emulsions, as M8 crystallizes first as fibers at 120 °C, followed by SFW crystallization at 78 °C. Consequently, M8 emulsions stability was not improved. In the fluorescence micrographs of M8-30 and M8W-30, water droplets were surrounded by aggregated M8 fibers. The fluorescent intensity of fibers was higher in M8W-30, compared to M8-30, indicating that the presence of wax crystals enhanced the aggregation of M8 fibers around the droplets. This suggests the existence of co-operation or synergy between M8 fibers and SFW. But this synergy seems insufficient as interstitial spaces were seen within the aggregated fibers (highlighted and shown with arrows in Figure 8c,f). An increase in fiber aggregation or fluorescent intensity was seen in S8W-50 against S8-50 (Figure 9c,f). The synergy between SFW and S8 fibers seem effective, as the interstitial spaces were not seen in S8W-50 but in S8-50.

**Figure 10:**
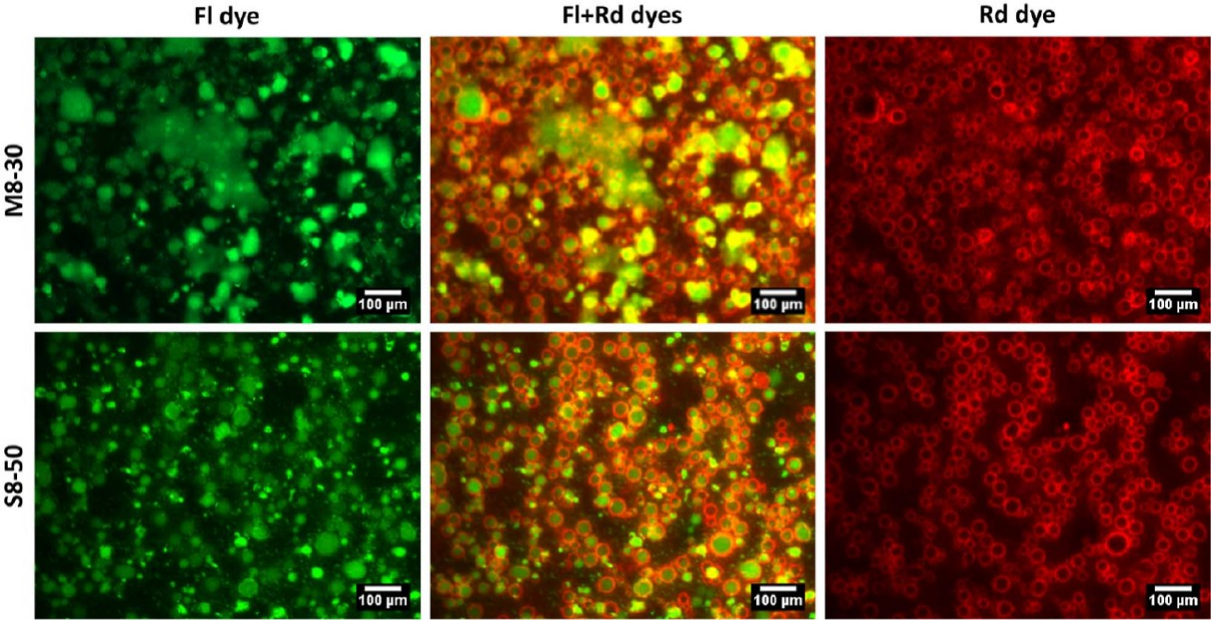
M8-30 and S8-50 emulsions with fluorescein and rhodamine B dyes.

To further elucidate the fiber aggregation, emulsions were prepared using two water-soluble dyes, fluorescein and rhodamine B. Figure 10 shows dispersed water droplets (green) with aggregated fibers (red) around them. In addition to water dispersion, M8-30 showed water patches, suggesting free water in the emulsion, which will eventually lead to phase separation. This study demonstrated that both gelators followed the same emulsion stabilization mechanism, i.e., solid particle network stabilization. This also proved that S8 is a more effective emulsifier than M8.

**Figure 11:**
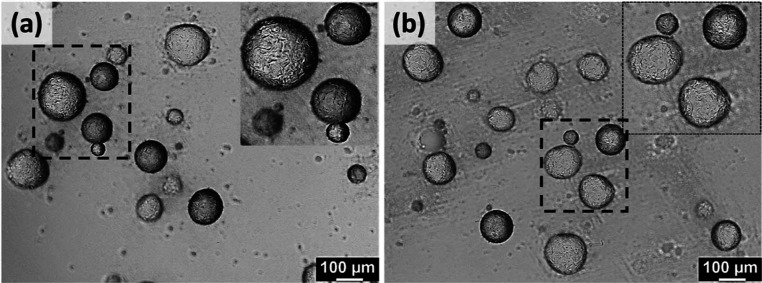
Diluted (a) S8-50 and (b) S8W-50 emulsions.

The efficiency of S8 fibers and their presence at the W/O interface were further confirmed by another simple study. 0.5 mL of S8-50 and S8W-50 were diluted in 5 mL of water. The surface adherence of S8 fibers was clearly seen in both S8 and S8W emulsions (highlighted in Figure 11). This confirms that S8 fibers are emulsifying and stabilizing the dispersed water in either the presence or absence of SFW. Stabilization of water droplets by S8 fibers in water may pave the way for the W/W emulsions. Microscopy upon M8 emulsions was not possible, as the hydrophobic M8 fibers quickly phase separated out upon dilution and dispersion no longer existed.

In this study, a systematic investigation was conducted to establish the emulsification efficiency and emulsification mechanism of molecular gelators. To understand the efficiency, emulsions were prepared by varying the gelator concentration (0.5 to 5% w/w) and water volume (10 to 65% w/v) (Figure 2). Both the gelators started forming emulsions at their respective MGCs; however, M8 and S8 emulsions remained stable up to three weeks and three months, respectively. The stereoisomeric structural difference between the gelators has affected their self-assembly, which in turn affected their amphiphilicity. M8 formed longer and more hydrophobic fibers than S8’s shorter, less hydrophobic fibers. Light and fluorescent microscopy revealed that S8 fibers are more closely associated with the dispersed water droplets than M8 fibers. This kind of fiber aggregation was responsible for the stability of the S8 emulsions. Along with the emulsification mechanism, the effect of emulsifying parameters (shear, gelator crystallization regime) and sunflower wax on the stability of emulsions was also tested. Sheared oleogels did not form emulsions compared with the structured oleogels. The postcrystallization regime of the structured oleogels yielded stable emulsions. In the presence of SFW (1% w/v), significant improvement in the emulsification efficiency and stability was not observed in both kinds of emulsions.

## Conclusions and Future Work

This study advances the knowledge of the sugar fatty acyl molecular gelators by establishing their emulsifying nature based on stereoisomerism. This study proved that stereoisomerism greatly affects emulsification efficiency, along with oil structuring. The stereoisomeric difference between sorbitol and mannitol is responsible for the formation of different SAFiNs and the emulsification behavior. S8 was identified as a better emulsifier than M8. The short and less crystalline S8 fibers showed better interfacial activity than the long, crystalline M8 fibers. SFW showed synergetic action with S8 fibers while improving the emulsion stability. This kind of behavior was not seen with the M8 fibers. Based on the results, we envision that sorbitol dioctanoate has potential as an emulsifier in food and cosmetic applications.

In the future, a detailed investigation of the rheological, thermal, and textural properties of stable emulsions would be performed. Further, S8 emulsions have the potential to be used as controlled delivery vehicles for drugs, nutraceuticals, and/or bioactive agents. Supramolecular emulsions using molecular gelators will open new avenues in the field of interfacial science and may pave the way for the development of advanced formulations for food, pharmaceutical, and personal care industries.

## Supporting Information Available

Detailed description of the emulsions’ composition, vials of S8, M8 emulsions, brightfield, fluorescent micrographs of emulsions, and graphs depicting the size distribution analysis (PDF)
