# Supplementary material for: Investigating the Emulsifying Mechanism of Stereoisomeric Sugar Fatty Acyl Molecular Gelators
Source: Langmuir. 2024 Jun 27;40(27):13763–72. doi: 10.1021/acs.langmuir.3c03274 (PMC11238593; doi:10.1021/acs.langmuir.3c03274)
Supplement: Supplementary file 1 — la3c03274_si_001.pdf [file la3c03274_si_001.pdf]

Supporting Information

Investigating the Emulsifying Mechanism of Stereoisomeric Sugar Fatty Acyl Molecular Gelators

Sai Sateesh Sagiri[1†], Malick Samateh[1,2], George John[1,2,*]

[1]Department of Chemistry and Biochemistry, The City College of New York, 160 Convent Avenue, New York, NY 10031.

[2]Doctoral Program in Chemistry, The City University of New York, Graduate Center, New York, NY 10016.

[†]*Current Address*: Department of Biotechnology, Vel Tech Rangarajan Dr. Sagunthala R&D Institute of Science and Technology, Chennai, Tamil Nadu 600062, India

*Corresponding authors (John): gjohn@ccny.cuny.edu; (Sagiri): drsaisateeshsagiri@veltech.edu.in

Number of pages: 12

Number of figures: 12

Number of schemes: 0

Number of tables: 2

Table S1: Composition of M8 and S8 emulsions

Sample	Gelator % (w/v)	Water % (v/v)	Stability	Sample	Gelator % (w/v)	Water % (v/v)	SFW % (w/v)	Stability
M8-10	5	10	1 week	M8W-10	5	10	1	1 week
M8-20	5	20	1 week	M8W-20	5	20	1	1 week
M8-30	5	30	<1 week	M8W-30	5	30	1	1 week
				M8W-40	5	40	1	1 week
S8-10	5	10	3 months	S8W-10	5	10	1	3 months
S8-20	5	20	3 months	S8W-20	5	20	1	3 months
S8-30	5	30	3 months	S8W-30	5	30	1	3 months
S8-40	5	40	3 months	S8W-40	5	40	1	3 months
S8-50	5	50	3 months	S8W-50	5	50	1	3 months
S8-60	5	60	<1 month	S8W-60	5	60	1	3 months
				S8W-65	5	65	1	3 months

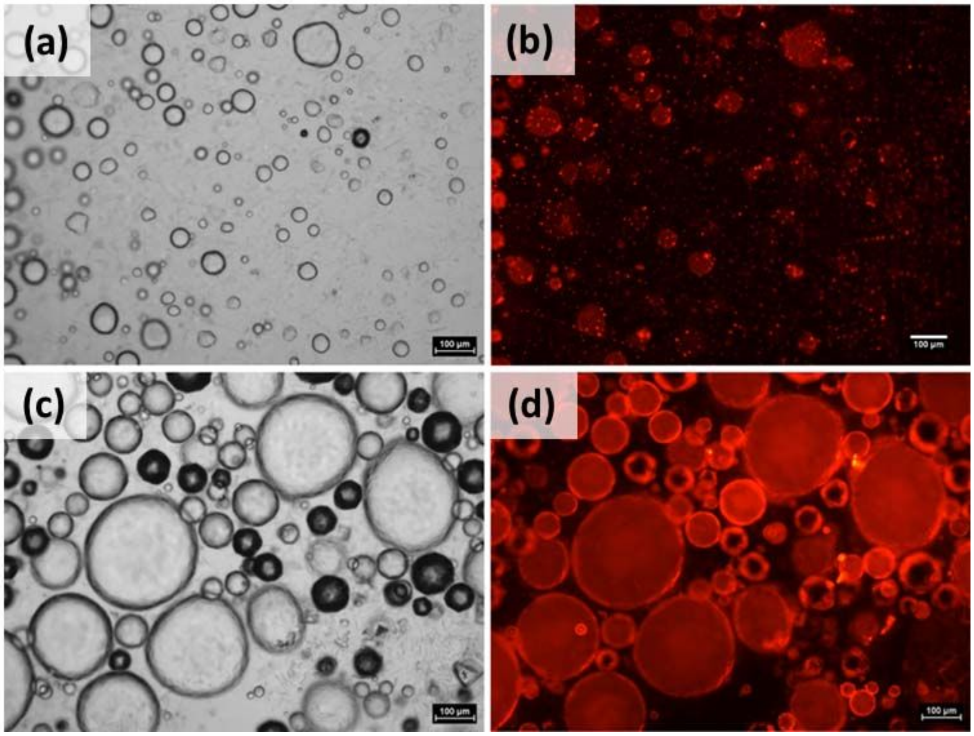

Figure S1: Brightfield and fluorescent images of M8 emulsions: (a), (b) when water was added to oleogel, and (c), (d) when oleogel was added to water.

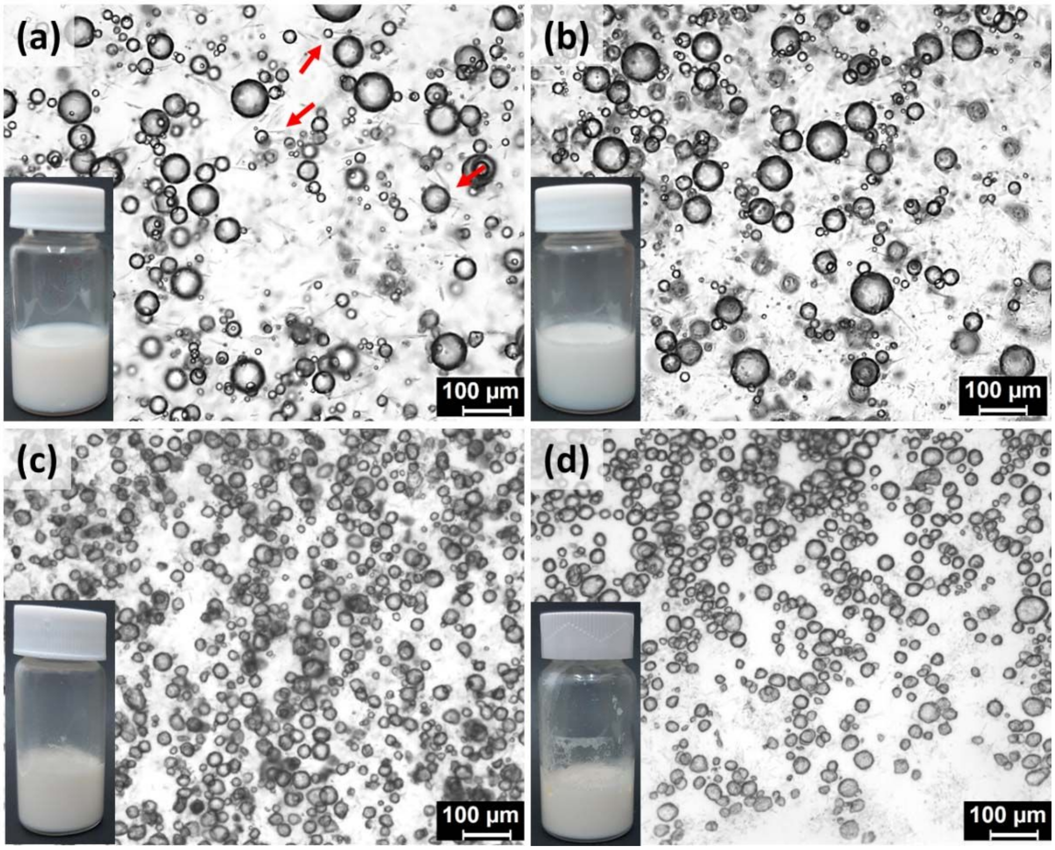

Figure S2: Microstructure of (a) M8 emulsion with pre-crystallization regime (b) M8 emulsion with post-crystallization regime, (c) S8 emulsion with pre-crystallization regime and (d) S8 emulsion with post-crystallization regime. Inserts in each micrograph are the corresponding vials of the emulsions.

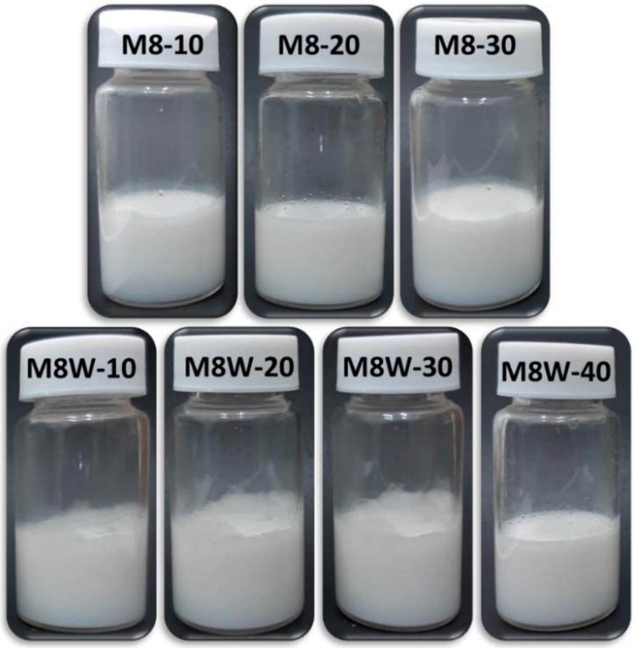

Figure S3: M8 and M8W emulsions after the preparation

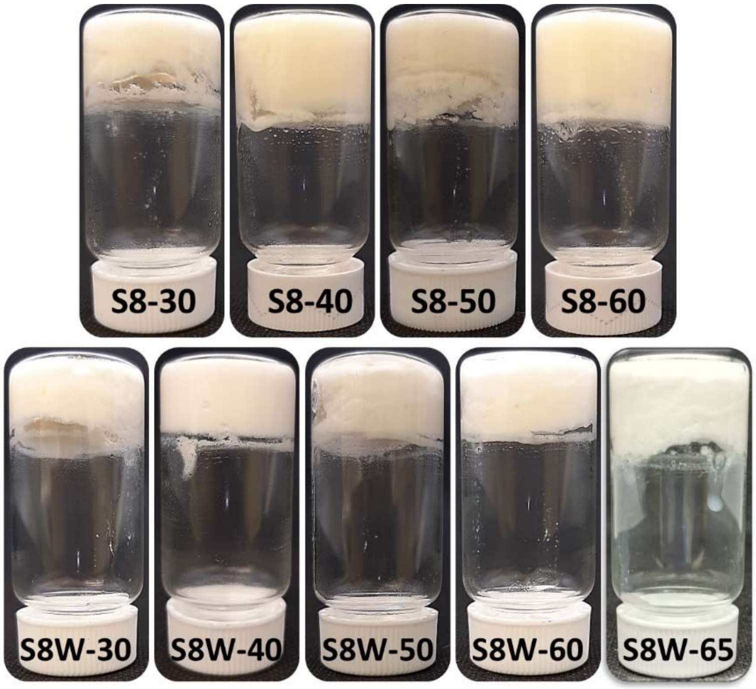

Figure S4: S8 and S8W emulsions after the preparation

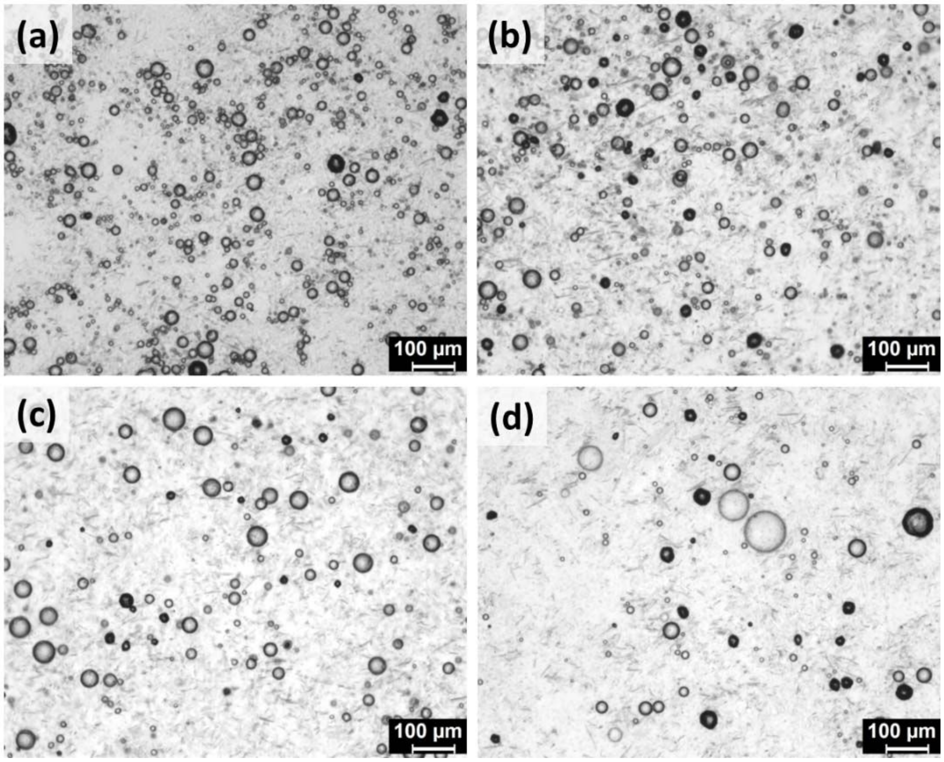

Figure S5: BFM images of (a) M8W-10, (b) M8W-20, (c) M8W-30 and (d) M8W-40 after the preparation.

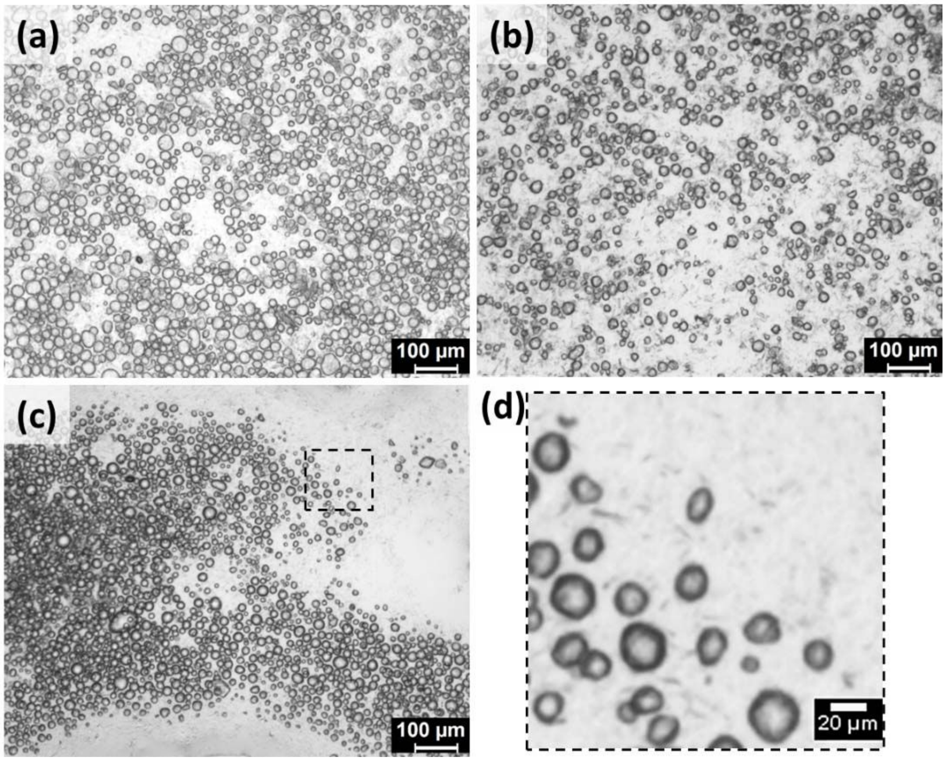

Figure S6: BFM images of (a) S8W-30, (b) S8W-40, (c) S8W-50 and (d) the magnified portion of S8W-50

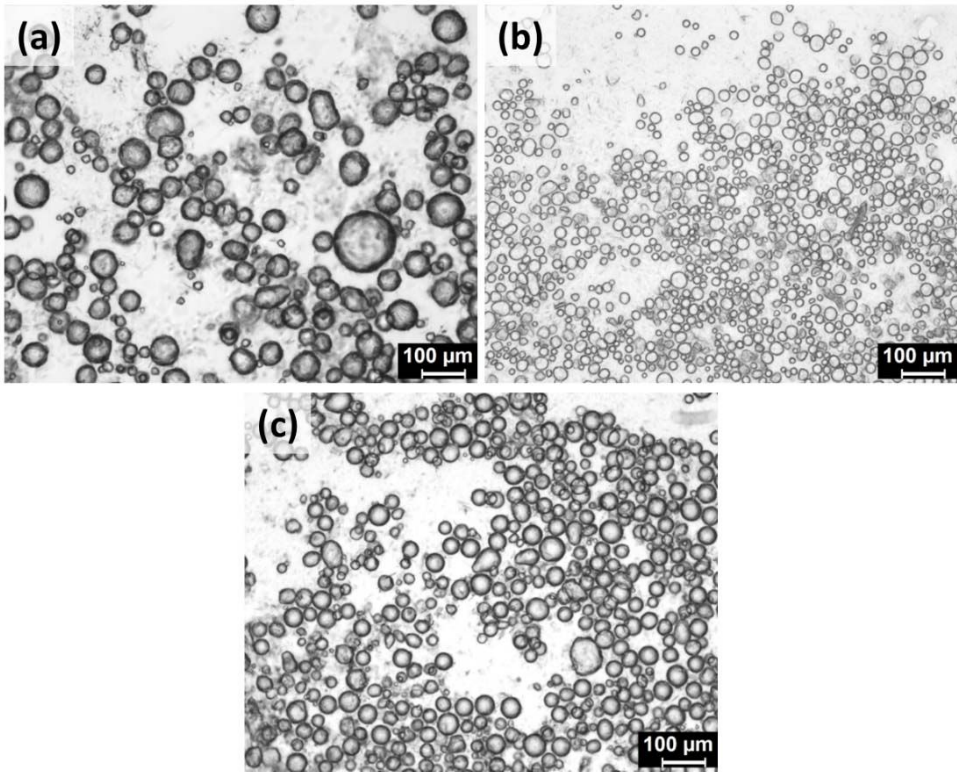

Figure S7: BFM images of (a) S8-60, (b) S8W-60, (c) S8W-65

Table S2: The PSD parameters of M8 and S8 emulsions

Samples	$D_{3,2}$ (µm)	$D_{v,0.5}$ (µm)	Span
M8-10	38.22	36.36	1.75
M8-20	44.43	40.0	1.72
M8-30	56.79	46.66	1.76
M8W-10	20.34	20.50	1.52
M8W-20	26.66	26.66	1.55
M8W-30	42.21	40.0	1.73
M8W-40	55.64	46.97	1.903
S8-30	20.59	22.22	0.91
S8-40	28.49	25.25	1.76
S8-50	32.51	28.28	1.03
S8-60	35.90	30.70	2.42
S8W-30	12.82	22.62	1.5
S8W-40	18.45	17.77	1.82
S8W-50	21.58	22.62	1.34
S8W-60	24.33	24.55	1.52
S8W-65	29.58	32.53	1.96
S8-50-1d	32.51	28.28	1.03
S8-50-30d	42.33	36.36	1.43
S8-50-60d	44.58	42.22	1.26
S8-50-90d	45.68	43.33	1.26
S8W-50-1d	21.58	22.62	1.34
S8W-50-30d	29.64	25.86	1.56
S8W-50-60d	30.78	26.36	1.59
S8W-50-90d	32.04	27.27	1.56

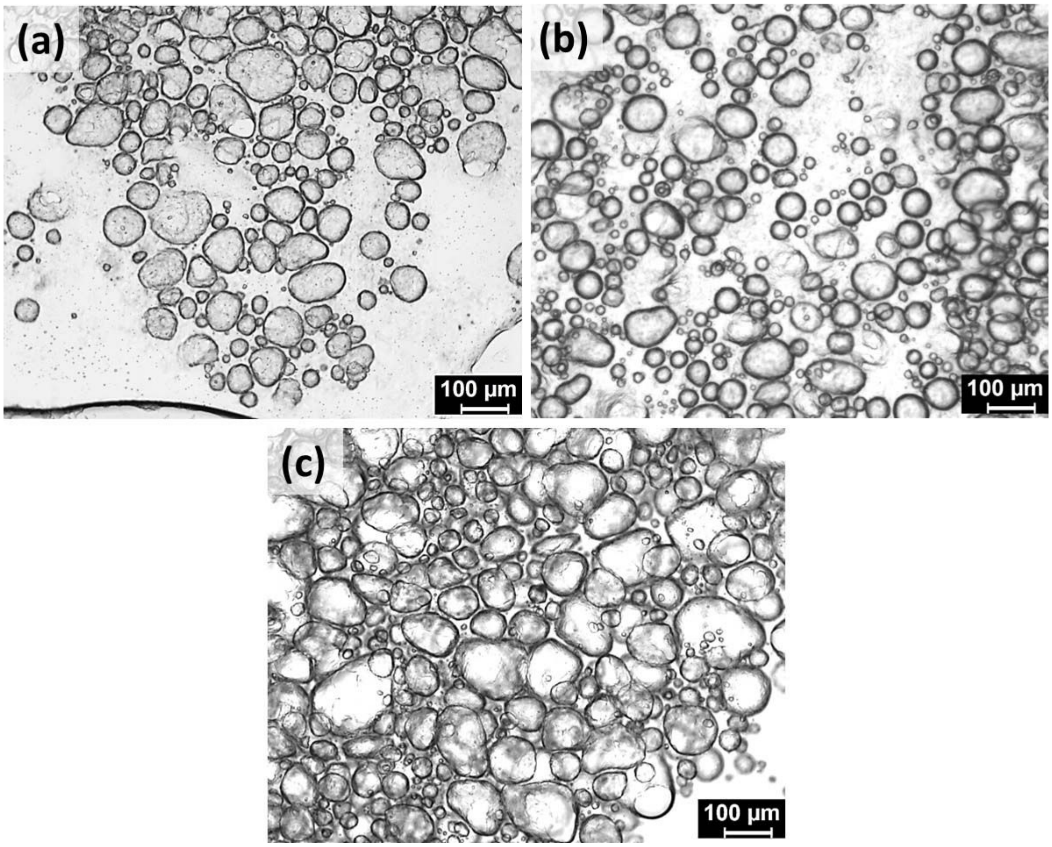

Figure S8: BFM images of (a) S8-60, (b) S8W-60 and (c) S8W-65

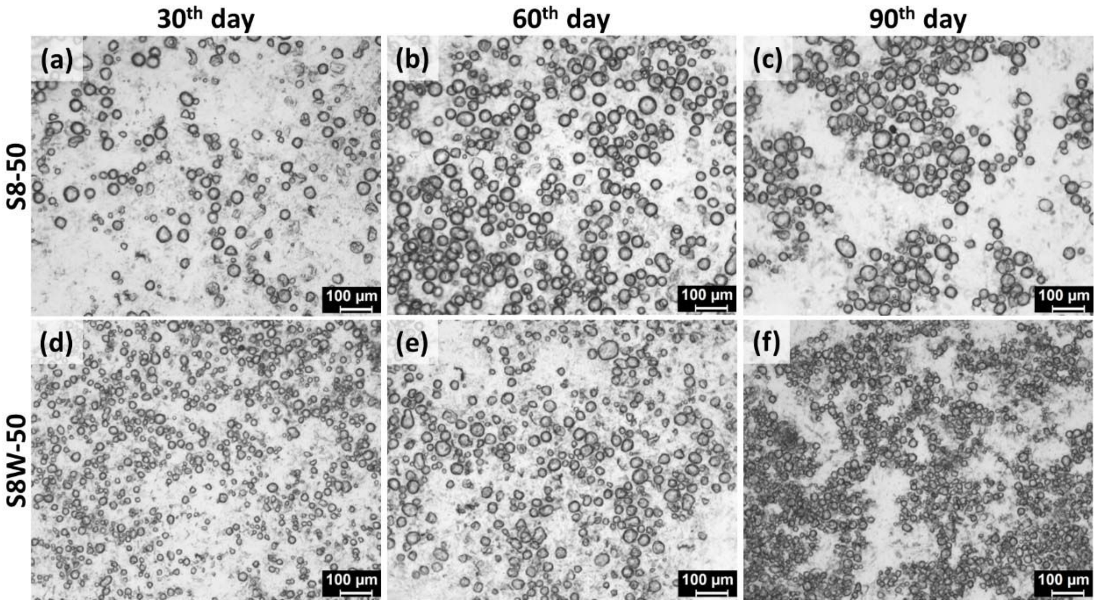

Figure S9: Periodical images of S8-50 and S8W-50 emulgels. S8-50 after (a) 30th day, (b) 60th day, (c) 90th day, and S8W-50 after (d) 30th day, (e) 60th day, (f) 90th day.

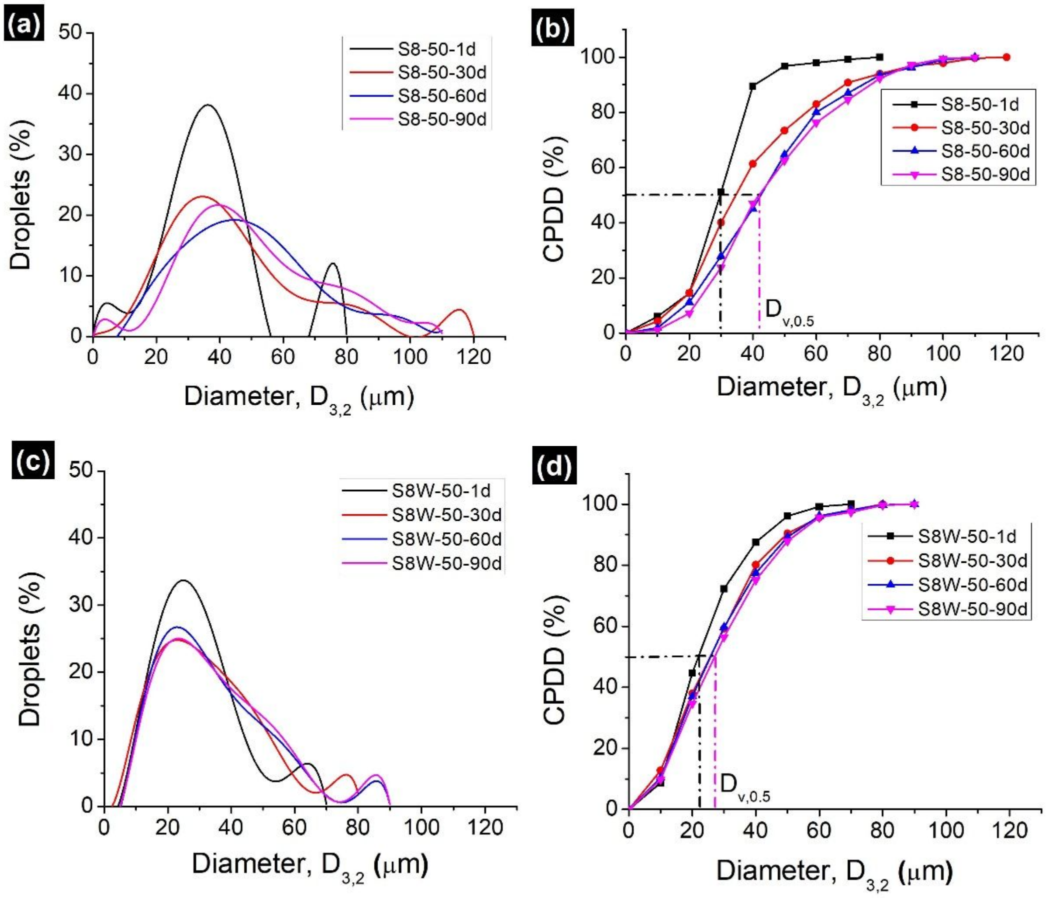

Figure S10: Day-wise SDA of (a), (b) S8-50 and (c), (d) S8W-50 emulgels

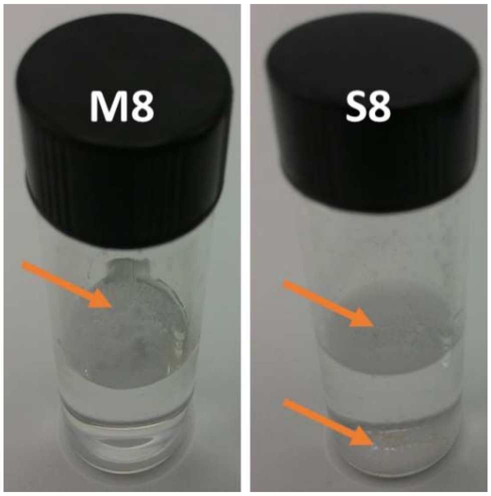

Figure S11: M8 and S8 in water

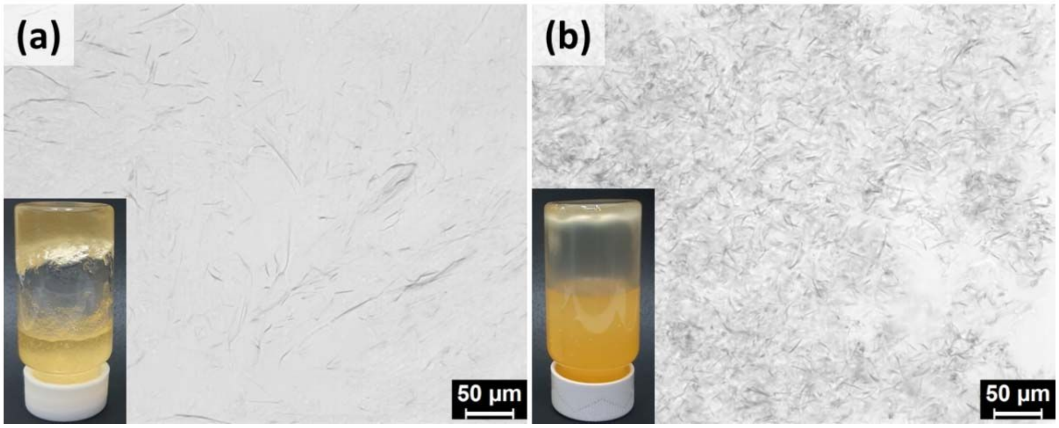

Figure S12: Microstructure and vials of sheared (a) M8 and (b) S8 oleogels
